# Systematic identification of target set-dependent activity cliffs

**DOI:** 10.4155/fsoa-2018-0089

**Published:** 2019-01-18

**Authors:** Huabin Hu, Dagmar Stumpfe, Jürgen Bajorath

**Affiliations:** 1 Department of Life Science Informatics, B-IT, LIMES Program Unit Chemical Biology & Medicinal Chemistry, Rheinische Friedrich-Wilhelms-Universität, Endenicher Allee 19c, D-53113 Bonn, Germany

**Keywords:** active and inactive compounds, activity cliffs, biological screening, matched molecular pairs, medicinal chemistry, open access data, potency, similarity

## Abstract

**Aim:**

Generating a knowledge base of new activity cliffs (ACs) defined on the basis of compound set-dependent potency distributions, also taking confirmed inactive compounds into account.

**Methodology:**

Different AC definitions, representations and search criteria were rationalized and applied.

**Data:**

For nearly 100 different target proteins, for which medicinal chemistry and biological screening data were available, target set-dependent ACs were identified. More than 20,000 target set-dependent ACs and associated information are made freely available.

**Limitations & next steps:**

As more compound data become available for new targets, the search for target set-dependent ACs, including confirmed inactive compounds will continue. Second-generation ACs will be subjected to systematic structure–activity relationship analysis.

## Lay abstract

In medicinal chemistry, activity cliffs can be rationalized just like geographical landscape features. Highly potent compounds occupy the top of mountains in activity landscapes and weakly potent compounds the surrounding valley floors. A small chemical change may be sufficient to push a highly potent compound off a steep cliff and transform it into a weakly potent or inactive valley floor compound. Hence, by studying activity cliffs, one can often understand which chemical modifications have large-magnitude effects on biological activity. We report a systematic search for a new type of activity cliffs using compound data from medicinal chemistry and biological screening.

## Graphical abstract

Shown are two activity cliffs involving the same highly potent compound (top). One cliff is formed with a weakly potent structural analog (bottom, left) and the other with an inactive analog (bottom, right). The analogs are active and confirmed inactive, respectively, against matrix metalloproteinase 14.

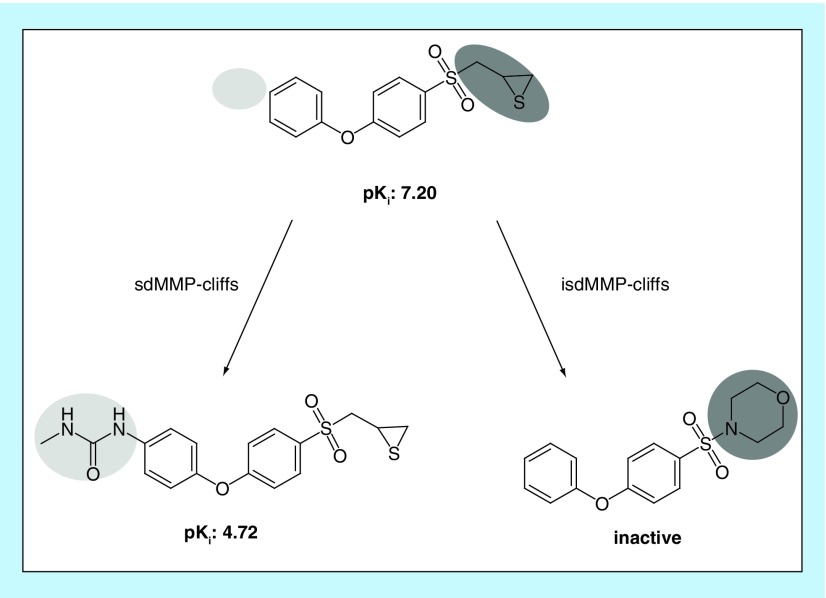

In medicinal chemistry, activity cliffs (ACs) are defined as pairs or groups of structurally similar compounds (analogs) having large differences in potency [1–3]. Accordingly, ACs are of high relevance for the exploration and exploitation of structure–activity relationships (SARs) [1–3]. For systematic AC analysis, the degree of compound similarity and the magnitude of potency differences required for AC formation must be clearly defined [1–3]. Similarity can be assessed on the basis of calculated molecular descriptors or, in a chemically more intuitive way, on the basis of structural relationships [2,3].

For the assessment of substructure-based similarity, that is, the presence or absence of well-defined substructures in compounds that are compared, the concept of matched molecular pairs (MMPs) [4,5] is particularly attractive and has been successfully applied to AC analysis [3,6]. An MMP is defined as a pair of compounds that only differ by a structural change at a single site [4,5]. This corresponds to the exchange of a pair of substructures such as a ring or another substituent (R-group). Following MMP terminology, this structural modification is termed a chemical transformation [5]. For MMP-based AC definition, transformation size-restricted MMPs (tsrMMPs) have been introduced [6] in which chemical transformations are limited in size to R-group replacements carried out in medicinal chemistry [6]. Accordingly, tsrMMPs consist of structural analogs, that is, compounds sharing the same core structure and carrying different R-groups at one or more sites. Another refinement of the MMP concept has been the generation of MMPs on the basis of retrosynthetic rules [7]. Thus, in this case, substitutions distinguishing compounds forming an MMP result from standard chemical reactions, leading to the introduction of retrosynthetic MMPs (RMMPs) [7].

Compound potency is usually assessed by determining (assay-dependent) pIC_50_ or (assay-independent) equilibrium constants (pK_i_ values). For AC analysis, equilibrium constants are strongly preferred to avoid the use of assay-dependent readouts when determining potency differences. An at least 100-fold difference in potency (two orders of magnitude) has often been applied as a general threshold for AC formation [1,2].

Typically, a search for ACs is carried out in different target sets [8], that is, sets of compounds with reported activity against individual targets (also termed compound activity classes). In global search calculations, constant similarity and potency difference criteria have been applied to identify ACs across different target sets [1,2]. For example, our preferred AC criteria include the formation of a tsrMMP or transformation size-restricted RMMP (tsrRMMP) and a potency difference between MMP forming compounds of at least two orders of magnitude, resulting in MMP-cliffs [6] or RMMP-cliffs [8].

However, generally applied potency difference thresholds do not take into account that potency value distributions significantly vary across target sets [8], depending on the nature of target–ligand interactions. This intrinsically limits the applicability of predefined constant potency difference thresholds for AC formation. Accordingly, ACs should best be defined in a target set-dependent manner by determining set-specific potency value distributions and setting set-dependent potency difference thresholds [8,9]. These variable thresholds account more accurately for target set-specific potency value distributions than generally applied constant thresholds.

The introduction of set-dependent potency difference thresholds as an extension of the AC concept requires additional analysis. First, target sets with large potency variations must be identified and prioritized [8]. This is the case because for target sets with narrow (‘flat’) potency value distributions, the determination of difference thresholds and definition of ACs is not meaningful and should be avoided. Second, for qualifying target sets, statistically significant potency differences between structural analogs must be determined [9]. MMPs with most significant potency differences then represent target set-specific MMP-cliffs.

Originally, ACs have exclusively been studied by comparing active compounds (sharing the same biological activity) [1,2]. However, as a further extension of the AC concept, compounds that are inactive against a given target might also be taken into consideration [10].

ACs formed by potent compounds and analogs that are experimentally confirmed to be inactive against the same target are also SAR-informative and increase the AC knowledge base.

In this Data Note, we report the results of a systematic search for target set-dependent MMP-cliffs and RMMP-cliffs, taking confirmed inactive compounds into account. As a part of our study, we have generated a large database of second-generation ACs that is described in detail and made freely available.

## Methodology

### Matched molecular pairs

MMPs were generated by systematic computational fragmentation of exocyclic single bonds in compounds according to Hussain and Rea [5]. To avoid the generation of MMPs with replacements of core fragments, which are often not regarded as ACs from a chemical perspective, only single-cut fragmentation [5] was carried out. For the generation of RMMPs, random bond fragmentation was replaced by fragmentation according to retrosynthetic rules [7], which typically reduces the number of MMPs. Thus, RMMPs are a subset of MMPs. In a tsrMMP [6] and tsrRMMP (abbreviated tsr(R)MMP in the following), the identical part of the two molecules (also called core structure) must at least be twice the size of the exchanged substructures. In addition, the difference in size between the exchanged substructures is limited to at most eight heavy atoms and a substructure is not allowed to contain more than13 heavy atoms [6]. This restricts transformations to R-groups replacements typically carried out in medicinal chemistry.

### Activity cliffs

Target set-dependent ACs were generated by applying the MMP-based definition taking confirmed inactive compounds into account. For the unambiguous definition of ACs, a similarity criterion and potency difference criterion must be clearly defined.

#### Definition of MMP-cliffs

In the original definition of MMP-cliffs, the following criteria were applied [6]:

- Similarity: formation of a tsrMMP.
- Potency difference: at least two orders of magnitude (100-fold) on the basis of equilibrium constants (K_i_ values).

This potency difference criterion was generally applied to all target sets, without considering set-specific potency value distributions.

#### Definition of target set-dependent MMP-cliffs & RMMP-cliffs

Second-generation MMP-cliffs and RMMP-cliffs (abbreviated [R]MMP-cliffs in the following) were defined by applying a constant similarity and variable target set-dependent potency difference criterion according to [9]:

- Similarity: formation of a tsr(R)MMP.
- Potency difference: set to the mean plus two standard deviations (sigma) of the distribution of potency differences encoded by all tsr(R)MMPs found in a given target set.

Hence, tsr(R)MMPs with statistically most significant potency differences within a given target set were classified as second-generation (target set-dependent) ACs (sd(R)MMP-cliffs). By definition, sd(R)MMP-cliffs were exclusively formed by active compounds. Only target sets with statistically significant potency variations according to [8] were considered as a source of these ACs.

#### Target set-dependent activity cliffs including inactive compounds

A search for additional (R)MMP-cliffs was carried out by considering structural analogs of potent target set compounds that were inactive in screening assays by applying the following criteria:

- Similarity: formation of a tsr(R)MMP by a potent compound and an inactive analog.
- Potency: target set compound with ‘AC relevant potency’ (see below).

For ACs involving an inactive compound, no potency difference criterion is applicable. Rather, for an active compound, an AC-relevant potency level must be defined. To avoid the formation of ‘pseudo ACs’ formed by weakly potent and inactive compounds, which would be irrelevant for SAR analysis, compounds with AC-relevant potency (ACRP_CPDs) were determined as follows:

- For a given target set, all sd(R)MMP-cliffs were identified.
- From each sd(R)MMP-cliff, the highly potent AC partner was extracted.
- The median of the potency distribution of unique highly potent AC partners was determined.
- Target set compounds with a potency equal to or greater than the median were selected as ACRP_CPDs.

**Figure 1.**
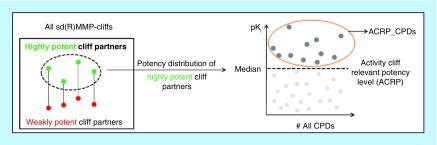
**Activity cliff-relevant compound potency.** The determination of the target set-dependent AC-relevant potency threshold is illustrated. From all sd(R)MMP-cliffs obtained for a given target set, highly potent cliff partners are extracted and their potency distribution is monitored. The median value of the distribution is determined (thick dashed horizontal line) and defines the AC-relevant potency threshold. All target set compounds exceeding this threshold are classified as ACRP_CPDs and systematically combined with available inactive compounds to yield isd(R)MMP-cliffs. AC: Activity cliff; ACRP_CPDs: Compounds with activity cliff-relevant potency; MMP: Matched molecular pair; RMMP: Retrosynthetic matched molecular pair; sd(R)MMP-cliff: Target set-dependent RMMP- or MMP-cliff.

The potency criterion for selecting ACRP_CPDs is illustrated in Figure 1. This criterion ensures that ACs involving inactive analogs are only formed with potent compounds. An sd(R)MMP-cliff involving an inactive analog is designated inactive analog sd(R)MMP-cliff (isd(R)MMP-cliff). In Table 1, different MMP-based AC definitions are summarized and explained. The same criteria apply for RMMP-cliffs, which represent a subset of all MMP-cliffs. This is the case because bond fragmentation according to retrosynthetic rules is covered by systematic random fragmentation leading to MMP formation.

**Table 1.** **Different matched molecular pair-based activity cliff definitions.**

**Activity cliff definition**	**Structural similarity**	**Potency difference**	**Cliff compound origin**
Original MMP-cliff	Transformation size-restricted matched molecular pair (tsrMMP)	≤ two orders of magnitude (Δpki2)	ChEMBL

sd MMP-cliff		Target-set dependent potency difference (Δpki0.9-2.7)

isd MMP-cliff		Pair of ACRP ChEMBL and inactive PubChem compound (no Δpki)	ChEMBL and PubChem

**Figure 2.**
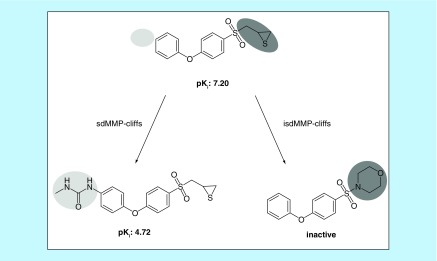
**Coordinated matched molecular pair-cliffs.** Shown are two MMP-cliffs involving the same highly potent compound. One MMP-cliff is formed with a weakly potent analog from the same target set, representing an sdMMP-cliff, and the other with an inactive analog from an assay for the same target, representing an isdMMP-cliff. isdMMP-cliff: Target set-dependent MMP-cliff with inactive analog; MMP: Matched molecular pair; sdMMP-cliff: Target set-dependent MMP-cliff.

The formation of an sdMMP- and isdMMP-cliff involving the same highly potent AC partner compound is illustrated in Figure 2.

### Active & inactive source compounds

As a source of compounds that were active or inactive against the same target, ChEMBL [11] and PubChem BioAssays [12] were used, respectively. ChEMBL and PubChem represent the major public repositories for compounds and activity data from medicinal chemistry and biological screening, respectively.

#### Compound activity data

From ChEMBL release 24.1, compounds with available high-confidence activity data were collected. Thus, only compounds involved in direct interactions (ChEMBL type ‘D’) with human targets at the highest level of assay confidence (assay confidence score 9) were selected. Furthermore, only numerically specified K_i_ values were considered as potency measurements (recorded in logarithmic form as pK_i_ values). Qualifying compounds were organized in target sets.

#### Inactive compounds

For targets with qualifying active compounds, a search was carried out in PubChem BioAssays. If primary and/or confirmatory screening assays for the same target were found, inactive compounds from these assays were collected. Compounds that are inactive in primary or confirmatory assays are designated ‘inactive’ in PubChem's assay records. Inactive compounds were directly selected from PubChem BioAssays because subsets of inactive compounds available in ChEMBL exclusively originate from PubChem.

### Identification of activity cliffs

sd(R)MMP-cliffs and isd(R)MMP-cliffs were systematically identified by applying the following protocol:

- Target sets for which inactive compounds were available were selected.
- For ChEMBL compounds, all sd(R)MMP-cliffs were identified.
- On the basis of sd(R)MMP-cliffs, ACRP_CPDs were defined.
- For each target set, a systematic search for tsr(R)MMPs involving ACRP_CPDs and inactive compounds was carried out.
- Target sets yielding isd(R)MMP-cliffs were identified.

## Data

### Search results summary

Table 2 reports the results of our search for target set-dependent ACs. From ChEMBL, 915 qualifying target sets were obtained, 525 of which contained statistically significant potency variations according to [8], giving rise to the formation of ACs. For these sets, MMPs and RMMPs were calculated, and 257 and 199 sets were selected that yielded at least 100 MMPs and RMMPs, respectively. The target sets contained 93 (MMP) and 73 (RMMP) sets for which inactive compounds were identified in PubChem. From these sets, sd(R)MMP-cliffs were extracted, yielding 13,546 sdMMP- and 7995 sdRMMP-cliffs, respectively. For target sets for which inactive compounds were available, a search for isd(R)MMP-cliffs was carried out by systematically comparing ACRP_CPDs and inactive compounds on a per-set basis. The search yielded a total of 340 isdMMP-cliffs that originated from 21 target sets (Table 3) and 145 isdRMMP-cliffs from eight sets (Table 4).

**Table 2.** **Target set, compound and activity cliff statistics.**

**Number of target sets**	**MMP**	**RMMP**
Total	915	915

Statistically relevant intraset potency variations	525	525

≥100 (R)MMPs	257	199

≥1 inactive PubChem CPDs	93	73

With isd(R)MMP-cliffs	21	8

**Number of CPDs (unique)**		

ACRP_CPDs	9504	8898

Inactive CPDs	479,774	446,367

**Number of ACs (target-based)**		

sd(R)MMP-cliffs	13,546	7995

isd(R)MMP-cliffs	340	145

Reported are qualifying target sets and compounds from which sd(R)MMP- and isd(R)MMP-cliffs were extracted. Statistics are provided for MMPs and RMMPs.

AC: Activity cliff; ACRP_CPDs: Compounds with activity cliff-relevant potency; CPD: Compound; isd(R)MMP-cliff: Target set-dependent RMMP- or MMP-cliff involving an inactive analog; MMP: Matched molecular pair; (R)MMP: (Retrosynthetic) MMP; sd(R)MMP-cliff: Target set-dependent RMMP- or MMP-cliff.

**Table 3.** **Target sets with sdMMP- and isdMMP-cliffs.**

**ChEMBL ID**	**Target name**	**Number of ACs**	

		***sdMMP-cliffs***	***isdMMP-cliffs***
2035	Muscarinic acetylcholine receptor M5	16	96

216	Muscarinic acetylcholine receptor M1	25	67

217	Dopamine D2 receptor	1444	66

1821	Muscarinic acetylcholine receptor M4	11	23

210	β-2 adrenergic receptor	33	21

1889	Vasopressin V1a receptor	60	18

3869	Matrix metalloproteinase 14	13	11

234	Dopamine D3 receptor	1145	5

2288	Peptidyl-prolyl cis-trans isomerase NIMA-interacting 1	20	5

2954	Cathepsin S	108	4

4018	Neuropeptide Y receptor type 2	10	4

2971	Tyrosine-protein kinase JAK2	19	3

1947	Thyroid hormone receptor β-1	15	3

242	Estrogen receptor-β	16	3

206	Estrogen receptor-α	15	2

2487	β-amyloid A4 protein	8	2

214	Serotonin 1a (5-HT1a) receptor	324	2

237	κ-opioid receptor	750	2

5113	Orexin receptor 1	368	1

3837	Cathepsin L	118	1

1871	Androgen receptor	60	1

Sum	21	4578	340

The table reports the number of sdMMP- and isdMMP-cliffs for each of 21 target sets. ChEMBL target IDs and target names are given.

AC: Activity cliff; isdMMP-cliff: Target set-dependent MMP-cliff with inactive analog; sd-MMP-cliff: Target set-dependent MMP-cliff.

**Table 4.** **Target sets with sdRMMP- and isdRMMP-cliffs.**

**ChEMBL ID**	**Target name**	**Number of ACs**	

		***sdRMMP-cliffs***	***isdRMMP-cliffs***
217	Dopamine D2 receptor	1016	59

216	Muscarinic acetylcholine receptor M1	12	44

1889	Vasopressin V1a receptor	51	18

210	β-2 adrenergic receptor	25	17

214	Serotonin 1a (5-HT1a) receptor	221	2

234	Dopamine D3 receptor	725	2

242	Estrogen receptor-β	5	2

237	κ-opioid receptor	406	1

Sum	8	2461	145

The table reports the number of sdRMMP- and isdRMMP-cliffs for each of eight target sets. ChEMBL target IDs and target names are given.

AC: Activity cliff; isdRMMP-cliff: Target set-dependent RMMP-cliff with inactive analog; RMMP: Retrosynthetic matched molecular pair; sdRMMP-cliff: Target set-dependent RMMP-cliff.

### Network analysis

Although only comparably small numbers of isd(R)MMP-cliffs are currently available, they encode novel SAR information, as revealed by AC network analysis.

In AC networks, nodes represent cliff compounds and edges indicate the formation of ACs [13]. Two cliff compounds are connected in the network if they form an (R)MMP and exceed the target set-dependent potency difference criterion, that is, each edge represents the formation of a unique MMP-cliff. Series of structurally related compounds with varying potency that form multiple and overlapping cliffs are represented by disjoint clusters in AC networks. ACs forming clusters are termed coordinated ACs. Clusters of increasing size contain increasing numbers of coordinated ACs and are a major source of SAR information [13]. Different (disjoint) clusters in an AC network consist of distinct analog series that form coordinated ACs. Disjoint clusters are directly selected from the network.

**Figure 3.**
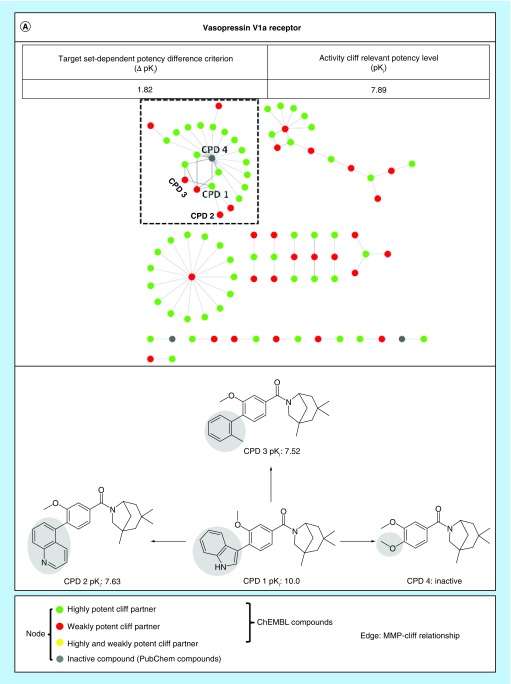

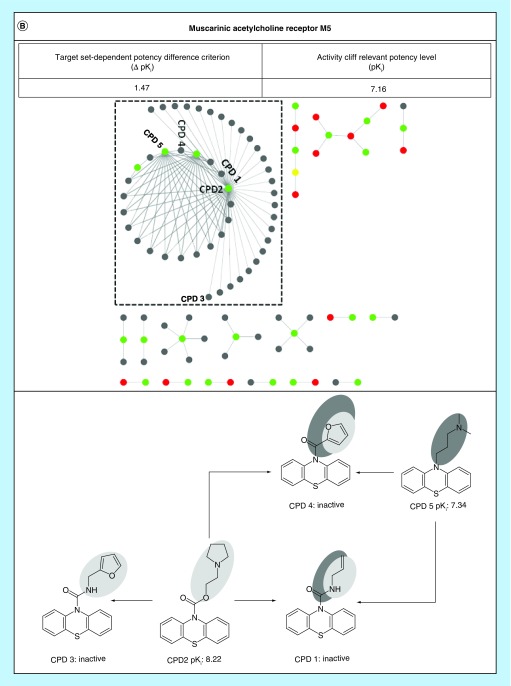
**Extended matched molecular pair-cliff networks.** Shown are exemplary AC networks for target sets for which inactive analogs of ACRP_CPDs were available. Nodes connected by an edge represent MMP-cliffs that are either isolated (single pair) or coordinated (leading to the formation of clusters with more than two nodes). Disjoint clusters represent different series of structurally related compounds involved in the formation of coordinated MMP-cliffs. The inclusion of inactive analogs results in the formation of isdMMP-cliffs (green and gray nodes connected by edges) that further increase the size of the network of sdMMP-cliffs (green, red and yellow nodes connected by edges) and lead to the formation of extended or new AC clusters. In compound structures, chemical modifications distinguishing AC compounds are highlighted. Target set: **(A)** Vasopressin V1a receptor (ChEMBL ID 1889); **(B)** Muscarinic acetylcholine receptor M5 (ID 2035). AC: Activity cliff; CPD: Compound; MMP: Matched molecular pair.

For each target set containing both sd(R)MMP- and isd(R)MMP-cliffs, two network were separately calculated for sd(R)MMP-cliffs as well as for the union of sd(R)MMP- and isd(R)MMP-cliffs and their cluster structures were compared. The addition of isdMMP-cliffs led to an increase in the number of AC clusters for 15 of 21 target sets, with one to nine new clusters per set, and to an extension of existing clusters, with one to four extended clusters per set. The addition of isdRMMP-cliffs yielded new clusters for six of eight target sets, with one to five clusters per set, and extended clusters for five of eight sets, with one to two clusters per set. New or extended clusters provide new SAR information. Figure 3 shows exemplary AC networks for different target sets (drawn with Cytoscape [14]).

The extension of existing AC clusters through inactive compounds leads to an increase in SAR information, even if only limited numbers of inactive compounds are added to the target sets, as revealed by network analysis.

#### Data deposition

All sdMMP-cliffs, isdMMP-cliffs, sdRMMP-cliff, and isdRMMP-cliffs we have identified are provided in two separate text files. For each of 93 and 73 target sets with sdMMP- and sdRMMP-cliffs, respectively, the ChEMBL ID, target name, corresponding PubChem assay IDs (AIDs) and AC-relevant potency threshold value are reported and ACs are provided including isdMMP-cliffs (for 21 of 93 sets) and isdRMMP-cliffs (eight of 73 sets). For each active AC compound, the ChEMBL ID, Simplified Molecular-Input Line-Entry System (SMILES) representation and potency value are given and for each inactive AC compound, the PubChem ID (CID) and SMILES representation. The data are made freely available as a deposition on the ZENODO open access platform [15].

## Limitations & next steps

Currently, there are no limitations in defining and searching for sd(R)MMP-cliffs and our analysis yielded a large population of these ACs. However, although assay data were available for 93 target sets with sdMMP-cliffs and 73 sets with sdRMMP-cliffs, isdMMP- and isdRMMP-cliffs were only identified for 21 and eight of these targets, respectively. Hence, MMP relationships between potent target set and inactive screening compounds were only infrequently detected. In this context, one should consider that inactive screening compounds are typically structurally diverse, which is likely to limit the number of ChEMBL/PubChem (R)MMPs.

Nonetheless, newly identified isd(R)MMP-cliffs are worth investigating from an SAR perspective, as nicely illustrated by comparing sdMMP-cliff networks and networks combining sdMMP- and isdMMP-cliffs. In combined networks, the formation of additional AC clusters was observed in almost all cases.

The number of isd(R)MMP-cliffs across different target sets and their target set coverage will likely further increase when more compounds and activity data become available. Hence, our search for second-generation ACs will continue and we intend to update our data deposition when significant numbers of new ACs are identified.

However, the database of target set-dependent ACs we make freely available already represents a rich source of SAR information. Hence, we will subject our current collection of second-generation ACs to systematic SAR analysis using different computational approaches.

**Executive summary**

**Background**

- The activity cliff (AC) concept is introduced.
- Generally defined and second-generation (target set-dependent) ACs are discussed.

**Methodology**

- Alternative AC definitions and search criteria are specified.
- Target set-dependent sd(R)MMP-cliffs (second-generation [target set-dependent] ACs) and corresponding isd(R)MMP-cliffs involving inactive analogs are introduced.

**Data**

- Results of a systematic search for these target set-dependent ACs are summarized.
- The relevance of sd(R)MMP-cliffs and smaller numbers of isd(R)MMP-cliffs for structure–activity relationship exploration is emphasized.
- An open access deposition containing >20,000 target set-dependent ACs is described.

**Limitations & next steps**

- Different frequencies of sd(R)MMP- and isd(R)MMP-cliffs are rationalized.
- The number of sd(R)MMP- and isd(R)MMP-cliffs is expected to increase.
- Second-generation ACs will be subjected to systematic structure–activity relationship analysis.
